# Enhancing Confidence in Psychiatry Assessment Performance: PDSA-Driven Improvements in a Medical Student OSCE Practice Course

**DOI:** 10.1192/bjo.2026.11155

**Published:** 2026-06-30

**Authors:** Theo Boardman-Pretty, Lisa Page

**Affiliations:** 1 Sussex Partnership NHS Foundation Trust, Brighton, United Kingdom; 2 Brighton & Sussex Medical School, Brighton, United Kingdom

## Abstract

**Aims:**

Objective Structured Clinical Examinations (OSCEs) are a major component of summative assessment for final year medical students. Psychiatry OSCEs may pose particular challenges, including time management, rapport building and completing complex tasks such as capacity and risk assessment. A psychiatry OSCE practice course has been running for final year students at Brighton & Sussex Medical School for several years, but its ongoing impact and alignment with learner needs had not been formally evaluated. This quality improvement project aimed to evaluate the course and iteratively improve it using PDSA methodology, to ensure continued effectiveness and relevance.

**Aim:** To assess the impact of a psychiatry OSCE practice course on students’ self-reported confidence and preparedness, and to use structured feedback to inform iterative improvements to course delivery.

**Methods:**

Pre- and post-course questionnaires were administered across multiple cohorts. Students rated confidence across relevant domains (psychiatry knowledge, psychiatry OSCE ability, preparedness for final OSCEs) and comparator domains (general OSCE ability, preparedness for final written exam) using Likert scales. Qualitative feedback was collected on perceived challenges, preferred case types and most useful elements of the course. Anonymised pre- and post-responses were paired using participant-generated identifiers. Following feedback, targeted changes were implemented between cohorts and evaluated through subsequent PDSA cycles.

**Results:**

Across cohorts, students reported increased confidence and preparedness following the course, with greater improvements in psychiatry-related domains than comparator domains. Confidence in psychiatry OSCE ability showed the greatest increase, improving by a mean of 1.4 points on a 5-point Likert scale. Examiner feedback and practice with repetition were consistently rated as the most valuable components.

Time management was the most frequently anticipated challenge and was in fact cited more often post-course. In response, two key changes were introduced for later cohorts: allowing up to two minutes for students to read OSCE instructions and choose when to begin, mirroring reasonable adjustments available in summative OSCEs; and providing a two-minute verbal warning before the end of the station. In cohorts following these changes, time management was less frequently cited as the most challenging aspect post-course.

**Conclusion:**

This QI project demonstrates that a psychiatry OSCE practice course improves student confidence, and that iterative, learner-informed adjustments enhance realism, inclusivity and educational value. Simple changes to timing and communication addressed key student concerns, particularly around time management and accessibility. Ongoing evaluation will aid refinement and sustainability of the course. These findings may inform design of similar courses across other medical schools.

